# Adhesive-Enhanced Sternal Closure: Feasibility and Safety of Late Sternal Reentry

**DOI:** 10.1155/2017/8605313

**Published:** 2017-05-29

**Authors:** Aaron J. Spooner, Holly E. M. Mewhort, Lisa M. DiFrancesco, Paul W. M. Fedak

**Affiliations:** ^1^Section of Cardiac Surgery, Department of Cardiac Sciences, Libin Cardiovascular Institute of Alberta, Cumming School of Medicine, University of Calgary, Calgary, AB, Canada; ^2^Departments of Pathology & Laboratory Medicine and Oncology, The University of Calgary, Calgary, AB, Canada

## Abstract

This clinical case report describes sternal reentry performed years after adhesive-enhanced sternal closure using Kryptonite bone cement. This report provides novel data on the late effects of this innovation. We observed that sternal reentry is feasible and safe. The adhesive did not weaken from biodegradation over a period of several years. There was no evidence of adherence to adjacent soft tissues or other nonbony deep mediastinal structures. Surgeons who receive patients who require redoing cardiac surgery after adhesive-enhanced closure with Kryptonite can be reassured that sternal reentry is safe and feasible.

## 1. Introduction

Simple wire cerclage remains the standard method of sternal closure following median sternotomy, despite risks of postoperative sternal displacement and dehiscence [1]. We previously described augmentation of wire cerclage sternal closure using “Kryptonite” bone cement (Doctors Research Group Inc.; Southbury, CT) in an effort to reduce the risk of sternal nonunion and accelerate postoperative recovery [2–4]. These findings have been further reproduced in the literature and the use of Kryptonite for sternal closure has been associated with reduced postoperative pain, accelerated sternal healing, and improved quality of life [5]. The early clinical results were encouraging but the late effects of adhesive-enhanced sternal closure were unknown.

## 2. Case Presentation

**Figure 1:**
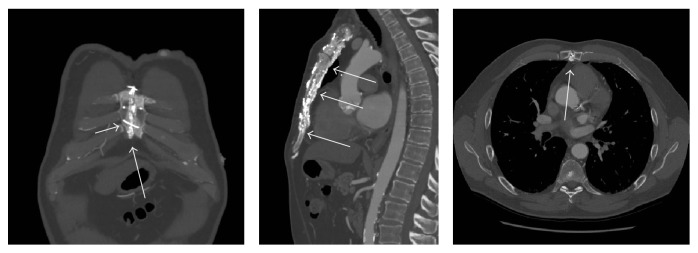
CT angiography 6 years post-CABG demonstrating sternal closure with wire cerclage (short arrow) and Kryptonite bone cement (long arrows).

A 67-year-old male presented with progressive symptoms of shortness of breath on exertion and presyncope. Investigations revealed newly diagnosed severe aortic stenosis, with a peak gradient of 93 mmHg, mean gradient of 45 mmHg, and an aortic valve area of 0.8 cm^2^ by continuity equation. The patient had prior coronary artery bypass grafting (CABG) 6 years priorly, at which time the median sternotomy was closed with wire cerclage augmented with “Kryptonite” bone cement. The technique of adhesive-enhanced sternal closure was described previously [4]. In preparation for surgical aortic valve replacement (SAVR), CT angiography was performed revealing extensive heterotopic ossification along the posterior margin of the prior sternotomy (Figure 1).

**Figure 2:**
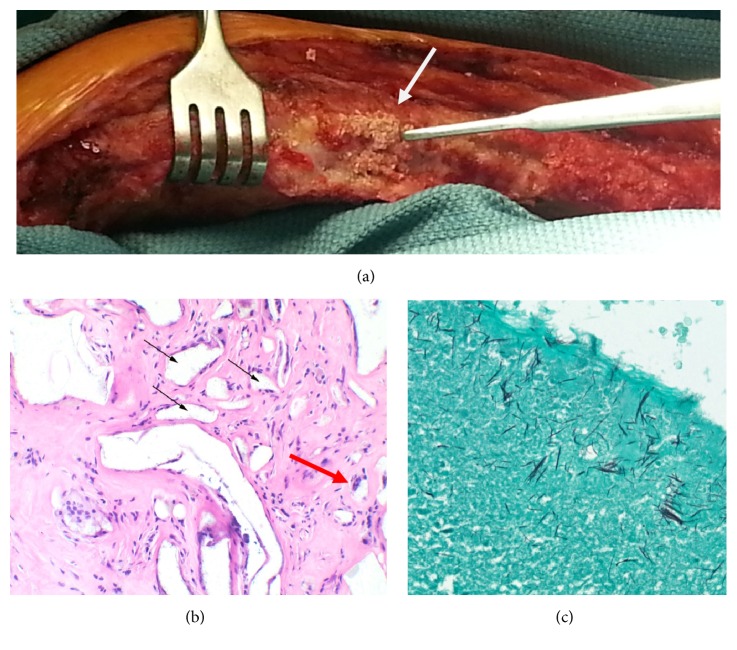
An intraoperative image demonstrating the appearance of Kryptonite (arrow) as it is peeled away from the cut-edge of the sternum after sternotomy (a). Hematoxylin and eosin staining demonstrating Kryptonite (clear granular filled “spaces” within the fibroconnective tissue; black arrows) and foreign body type giant cells (red arrow; 20x) (b). Grocott silver staining highlighting the fibrillary structure of Kryptonite (10x) (c).

A standard redo median sternotomy was performed to obtain access for surgical AVR. The sternum was found to be stable and intact with good callous formation along its length (Figure 2(a)). The midline sternotomy was reopened using an oscillating saw and heavy Mayo scissors without complication or difficulty, comparable to an average redo resternotomy. The tissue immediately adjacent to the posterior sternal table was areolar and easily dissected along the length of the sternum using electrocautery. The Kryptonite adhesive was visible as an opaque white solid material along the edges of the periosteum (Figure 2(a)) and was dissected away from the bone using DeBakey forceps and an oscillating saw. The adhesive was strongly adherent to the bone surfaces and well integrated. There was no gross evidence of inflammation or adhesions to adjacent nonbony tissues. There was no evidence of migration of the adhesive or substantial degradation or resorption. The underlying bone was in good condition. A small piece of the Kryptonite/sternal interface was removed and sent for histology. The remainder of the procedure was performed and standard wire cerclage was easily performed to reapproximate the sternum. Reapproximation was not supported by Kryptonite due to institutional availability at the time of the reoperation.

The histopathologic sample was fixed in 10% buffered formalin and subsequently decalcified in 10% formic acid. Following this, 6-micron sections were stained with hematoxylin and eosin (H&E) and examined under light microscopy. Histology revealed benign bone, bone marrow, cartilage, and adjacent fibroconnective tissue, as well as collections of nonpolarizable foreign material (“Kryptonite” adhesive). The latter did not stain with H&E, but had a yellow-brown, glassy, and granular appearance. The Kryptonite was arranged in round and ovoid circumscribed clusters, occasionally surrounded by giant cells, which appeared to engulf the Kryptonite (Figure 2(b)). Silver staining highlighted the microscopic fibrillary structure of the Kryptonite (Figure 2(c)).

## 3. Discussion

Over the past decade we pioneered an innovative new approach to enhance sternal closure in cardiac surgery patients. We developed a simple and efficient surgical technique whereby a novel biocompatible adhesive polymer is added to the sternal edges at the time of sternal closure. Within hours, the bone-like adhesive adds mechanical strength and stabilizes the reapproximated sternal edges. Pilot clinical data suggest that sternal bone healing is accelerated and postoperative recovery is improved [3]; findings have been reproduced by other independent groups [5]. The Kryptonite bone cement has also been utilized in neurosurgical procedures involving the cranium and vertebrae with good results [6, 7]. While the innovation requires substantial further validation, we observed global enthusiasm from both surgeons and patients. Since our first report, surgeons in Canada, USA, Australia, and Europe have successfully performed adhesive-enhanced sternal closure. Adhesive-enhanced sternal closure has the potential to accelerate recovery after cardiac surgery and improve bone union resulting in improved patient outcomes.

To that end, a rigorous research strategy is imperative to determine mechanisms of benefit, optimal techniques of application, feasibility, and safety, and the nature and magnitude of the clinical benefits provided by use of adhesives to enhance sternal closure. The late effects of adhesive-enhanced sternal closure are unknown including the risks and feasibility of late sternal reentry. This clinical report provides specific data on such issues. We observed that (1) remote sternal reentry after adhesive-enhanced sternal closure is feasible and safe, (2) the adhesive did not appear to substantially weaken from biodegradation over a period of several years, and (3) there was no evidence of adherence to adjacent soft tissues or other nonbony deep mediastinal structures. The optimal characteristics of a bone adhesive to enhance sternal closure remains to be developed and validated.
